# Predictive Factors of Hemorrhage After Thrombolysis in Patients With Acute Ischemic Stroke

**DOI:** 10.3389/fneur.2020.551157

**Published:** 2020-11-03

**Authors:** Fan Sun, Heng Liu, Hui-xiao Fu, Cheng-bo Li, Xiao-jing Geng, Xiao-xuan Zhang, Jiang Zhu, Zheng Ma, Yan-jun Gao, Zhi-jie Dou

**Affiliations:** Neurology Department, Affiliated Hospital of Chengde Medical University, Chengde, China

**Keywords:** acute ischemic stroke, hemorrhagic transformation (HT), caveolin-1 (Cav-1), caveolin-2 (Cav-2), biomarkers

## Abstract

**Background:** Ischemic stroke has a poor prognosis and brings a ponderous burden on families and society. Hemorrhagic transformation (HT) after intravenous thrombolysis can increase the mortality of patients with ischemic stroke. Thus, finding new HT biomarkers to be applicable in clinical practice is of great importance.

**Methods:** The related risk factors were recruited for analysis, including smoking, drinking, hyperlipidemia, diabetes, anamnesis, and pathological indicators. Moreover, the relationship between serum levels of caveolin-1, caveolin-2, and HT after rt-PA treatment were also studied.

**Results:** We studied 306 patients with acute ischemic stroke treated with recombinant tissue type plasminogen activator (rt-PA) within 4.5 h of symptom onset. The results showed that Age ≥68 years, smoking, Atrial fibrillation, NIHSS score before thrombolysis ≥17, and systolic pressure 2 h after thrombolysis (mmHg) ≥149 increased the risks of HT after rt-PA administration. Remarkably, the concentration of caveolin-1 (ng/mL) ≤ 0.12 and caveolin-2 (ng/mL) ≤ 0.43 in serum increased the risks of HT after rt-PA administration.

**Conclusion:** Knowledge on the risk factors associated with HT after rt-PA treatment may help develop treatment strategies and reduce the risk of HT. Caveolin-1 and caveolin-2 can be predictors of HT after rt-PA administration. These findings provide evidence for future further investigations aimed to validate these biomarkers.

## Introduction

Ischemic stroke is a common disease with high morbidity and mortality. It often has a poor prognosis and brings increasing burden on families and society (1). Intravenous thrombolysis with recombinant tissue type plasminogen activator (rt-PA) is an effective approach to treat acute ischemic stroke. However, HT after intravenous thrombolysis can increase the mortality of patients with ischemic stroke (2). HT refers to incidental intracerebral hemorrhage after acute ischemic stroke, which is a common complication for patients with acute ischemic stroke (3). Parenchymal hematomas to small petechiae are all HT. The occurrent proportion of HT after acute ischemic stroke ranges from 8.5 to 30%, in which 2.1–9.4% are symptomatic HT (4–6).

Matrix metalloproteinase and cellular fibronectin, which are considered biomarkers of endothelial damage, can act as HT predictors after intravenous thrombolysis (7). These biomarkers in serum improve the prediction ability of HT. It is reported that the association of matrix metalloproteinase and cellular fibronectin can improve the predictive capacity of relevant HT, suggesting that the analysis of several biomarkers may be necessary to achieve the necessary accuracy for these biomarkers to be applicable (8). Thus, finding new biomarkers to be applicable in clinical practice is of great importance.

Caveolins, which are composed of caveolin-1, caveolin-2, caveolin-3, are structural proteins of caveolae (9). Caveolin-2 is mainly co-expressed with caveolin-1, whereas caveolin-3 is specifically expressed in skeletal muscle (10). Caveolin-1 and caveolin-2 can modulate the blood–brain barrier permeability of rats with cerebral ischemia and glioma (11–13). In addition, caveolin-1 can regulate endothelial permeability after ischemic stroke (14). The caveolin-1 level in serum is also an important HT predictor after rt-PA administration (15). However, the association between serum caveolin-2 levels and HT development after thrombolysis remains unknown. This study aimed to investigate whether or not serum caveolin-2 levels are associated with HT.

## Materials and Methods

The study was approved by the Human Ethics Committees of Chengde Medical College. Informed consent was obtained from the patients or their relatives.

Between January 2014 and June 2019 306 patients with acute ischemic stroke treated with rt-PA within 4.5 h from the affiliated Hospital of Chengde Medical College were prospectively included in this study. The patients with known inflammation, infectious tumor diseases, and non-availability of blood samples were excluded from this study.

On admission, demographic data, including history, comorbidity, medical treatment before admission, and pathological indicators were recorded. Blood samples for the analysis of caveolin-1 and aveolin-2 were obtained from patients on arrival at the hospital. Multimodal perfusion cranial computed tomography was performed before r-tPA administration, and a simple cranial CT was performed at 24–36 h for the evaluation of HT, which was classified as hemorrhagic infarction type 1 and 2, and parenchymal hemorrhage type 1 and 2. Blood samples were obtained from patients before rt-PA administration. Blood samples were then centrifuged, and serum was stored at −80°C until analysis. The investigators who were blinded to the study analyzed the clinical data.

SPSS 24 statistical software (Chicago, IL, USA) was used to support data analysis. Data were displayed as percent values and absolute numbers. Between-group differences were analyzed by Student's *t-*test, and chi-square test was employed for univariate analysis. Multivariate logistic regressions were used to analyze the relationship between HT and risk factors. *T-*test was used to evaluate the statistical effect of each factor in multivariate logistic regression and *f-*test was used to whole multivariate logistic regression. We establish the final multi-factor model by stepwise regression. *P* ≤ 0.05 was considered statistically significant. The ROC (Receiver operating characteristic) curve was used to evaluate the performance of the model.

## Results

### Univariate Analysis of HT After rt-PA Administration

Three hundred and six patients with acute ischemic stroke treated with rt-PA within 4.5 h from the affiliated Hospital of Chengde Medical College were prospectively included in this study. These patients were divided into two groups (hemorrhage group and non-hemorrhage group). There are 52 patients in hemorrhage group and 254 patients in non-hemorrhage group. The results of univariate analysis showed that old age, high body mass index, and smoking augmented the HT incidence rate (Table 1). Atrial fibrillation, high level of hypersensitive C-reactive protein, high level of uric acid, high NIHSS score before thrombolysis, and high systolic pressure 2 h after thrombolysis (especially more than 150 mmHg) also increased the risk of HT. Both the serum levels of caveolin-1 and caveolin-2 in hemorrhage group (0.14 ± 0.025 and 0.46 ± 0.043 ng/ml, respectively) are lower than in non-hemorrhage group (0.39 ± 0.046 and 0.47 ± 0.051 ng/ml, respectively). Thus, caveolin-1 and caveolin-2 were associated with HT after rt-PA administration.

**Table 1:** The univariate analysis comparing patients with and without HT.

	**Hemorrhage group (*n =* 52)**	**Non-hemorrhage group (*n =* 254)**	**t/Chi-square**	***P*-value**
**Basic Information**				
Gender			0.1032	0.748
Male	37 (0.71)	175 (0.69)		
Female	15 (0.29)	79 (0.31)		
Age	68.92 ± 7.41	65.42 ± 6.56	0.235	0.0007
BMI index	24.58 ± 3.21	22.26 ± 2.62	3.945	<0.0001
**Risk factor**				
Smoking	21 (0.40)	48 (0.19)	11.14	0.0007
Drinking	18 (0.35)	73 (0.29)	0.7131	0.3984
Hyperlipidemia	36 (0.69)	147 (0.58)	2.316	0.1281
Diabetes	34 (0.65)	138 (0.54)	1.346	0.246
**Anamnesis**				
Valvular disease	19 (0.37)	96 (0.38)	0.02906	0.8646
Atrial fibrillation	26 (0.50)	84 (0.33)	5.373	0.0205
**Pathological indicators**				
Systolic pressure before thrombolysis (mmHg)	146.86 ± 14.47	147.23 ± 13.71	0.1756	0.8607
Diastolic pressure before thrombolysis (mmHg)	91.56 ± 8.43	89.96 ± 7.86	1.321	0.1876
Blood glucose before thrombolysis (mmol/L)	7.49 ± 1.31	7.37 ± 1.25	0.6256	0.5321
Triglyceride before thrombolysis (mmol/L)	1.82 ± 0.41	1.79 ± 0.38	0.5117	0.6092
hsCRP before thrombolysis (mg /L)	3.87 ± 0.74	1.57 ± 0.36	33.81	<0.0001
HDL-C before thrombolysis (mmHg)	1.22 ± 0.41	1.25 ± 0.39	0.501	0.6168
NIHSS score before thrombolysis	18.68 ± 4.53	14.31 ± 3.65	7.532	<0.0001
Platelets before thrombolysis (×10^9^/L)	217.53 ± 53.39	213.43 ± 48.26	0.548	0.5841
Uric acid before thrombolysis (μmol/L)	358.97 ± 48.85	342.02 ± 45.97	2.397	0.0172
Systolic pressure 2 h after thrombolysis (mmHg)	157.56 ± 14.69	136.81 ± 15.25	8.994	<0.0001
Diastolic pressure 2 h after thrombolysis (mmHg)	89.18 ± 11.24	87.83 ± 9.48	0.9053	0.366
**Blood protein**				
Caveolin-1 (ng/ml)	0.14 ± 0.025	0.39 ± 0.046	38.02	<0.0001
Caveolin-2 (ng/ml)	0.46 ± 0.043	0.47 ± 0.051	9.863	<0.0001

### Multivariate Logistic Regression Analysis of HT After rt-PA Administration

**Figure 1:**
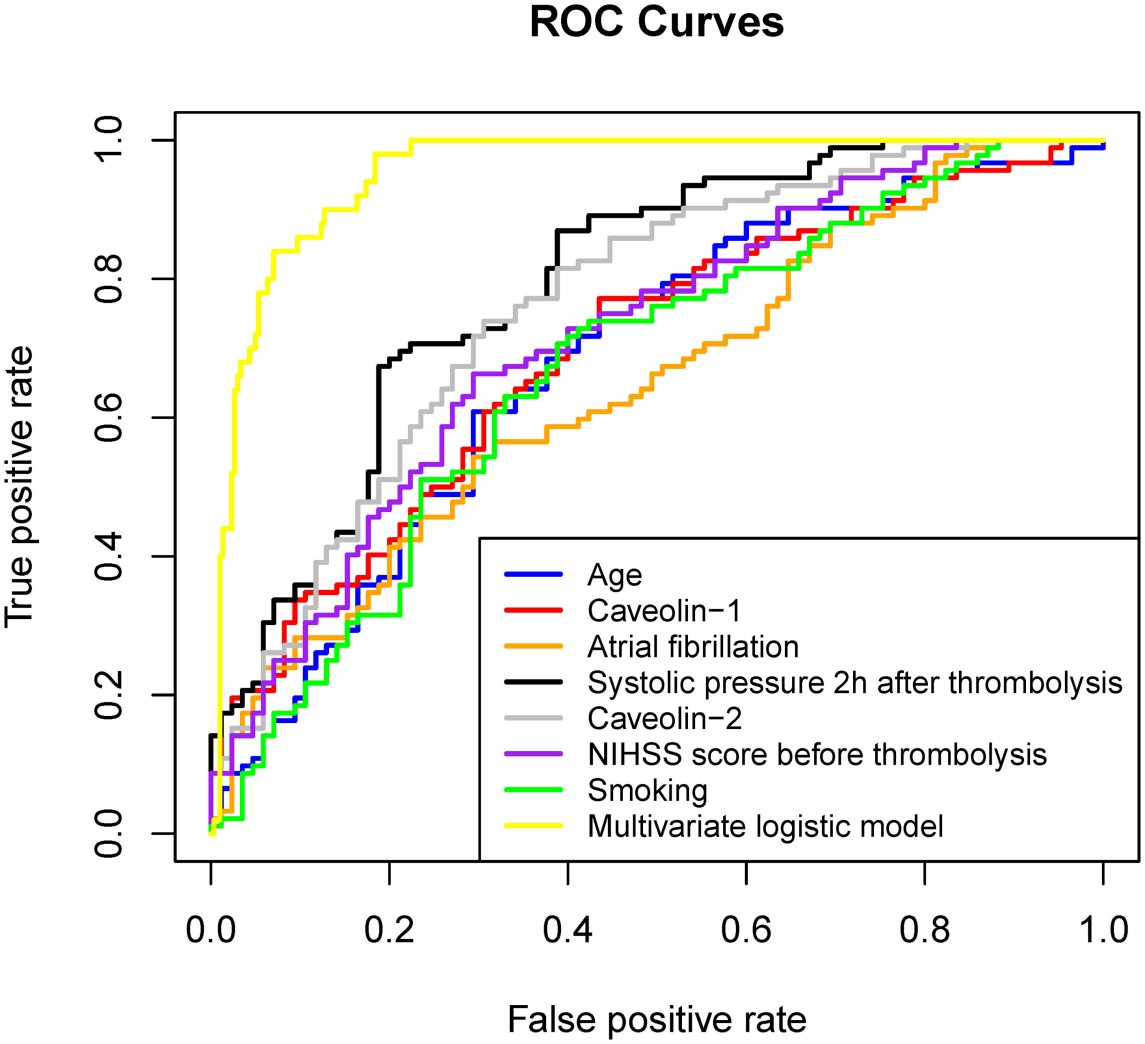
ROC curve of each factor in univariate logistic regression and multivariate logistic regression established by these factors.

Multivariate logistic regression analysis was performed by using HT as a response variable. Stepwise regression method made us to finalize the elements contained in the model. Age ≥68 years, smoking, Atrial fibrillation, NIHSS score before thrombolysis ≥17, and systolic pressure 2 h after thrombolysis (mmHg) ≥149 increased the risks of HT after rt-PA administration. Remarkably, the concentration of caveolin-1 (ng/mL) ≤ 0.12 and caveolin-2 (ng/mL) ≤ 0.43 in serum increased the risks of HT after rt-PA administration (Table 2). Figure 1 shows the ROC curve of each element in the univariate logistic regression and the ROC curve of the multiple logistic regression model (specific values of AUC were showed in Table 2). The AUC of the multiple logistic regression model is 0.954, which proves the reliability and rationality of the model.

**Table 2:** Multivariate logistic regression analysis of risk factors for HT.

**Index**	**OR(95% CI)**	***P*-value**	**AUC**
Age ≥ 68	1.835 (1.361–2.826)	0.024	0.692
Smoking	2.352 (2.174–3.580)	0.016	0.669
Atrial fibrillation	2.140 (1.492–2.453)	0.028	0.647
NIHSS score before thrombolysis ≥ 17	1.783 (1.156–3.247)	0.008	0.757
Systolic pressure 2 h after thrombolysis (mmHg)	4.251 (2.663–5.710)	0.004	0.794
Caveolin-1 (ng/ml)	1.972 (1.291–3.186)	0.031	0.700
Caveolin-2 (ng/ml)	2.759 (1.685–4.462)	0.005	0.762
Multivariate logistic model	7.317 (3.259–10.662)	0.001	0.954
Multivariate logistic model without Caveolin-1&2	5.272 (2.718–7.851)	0.003	0.844
Multivariate logistic model without Caveolin-1	6.635 (3.196–9.258)	0.001	0.911
Multivariate logistic model without Caveolin-2	6.348 (3.292–8.675)	0.002	0.883

**Figure 2:**
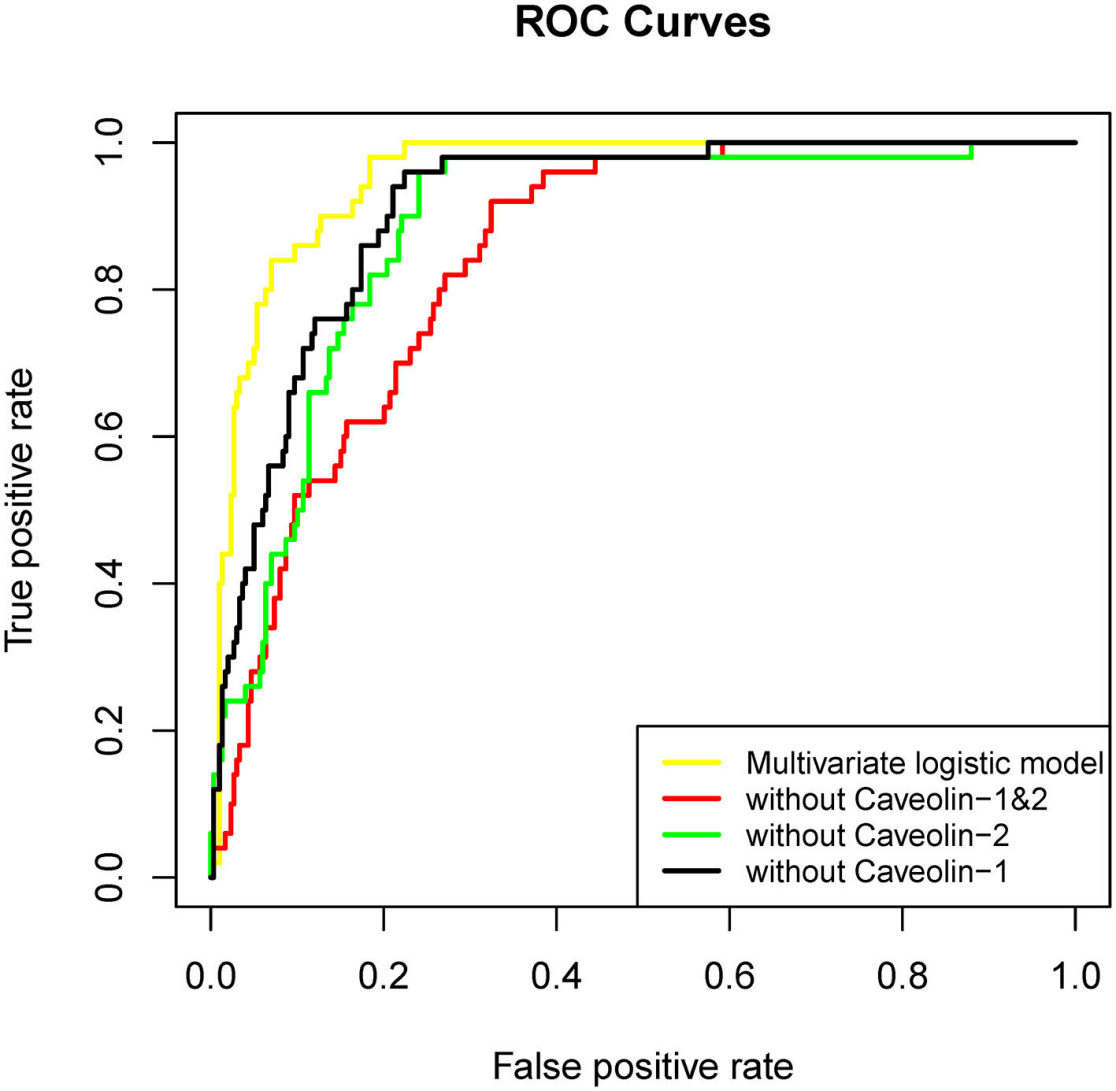
ROC curve of each factor in univariate logistic regression and multivariate logistic regression established by these factors (without caveolin-1&caveolin-2).

For demonstrating the contribution of caveolin-1 and caveolin-2 in predicting HT another 3 Multivariate logistic regression model were established. These three models were removed caveolin-1, caveolin-2, caveolin-1&caveolin-2 separately on the basis of the original variables. The results showed that AUC of the model without caveolin-1&caveolin-2 was 0.844. AUC of the models without caveolin-1 or caveolin-2 were 0.911 and 0.883, accordingly (Figure 2, Table 2). In another words, if the multivariate logistic regression model had losen variates, caveolin-1 and caveolin-2, it would have losen a fair share of reliability at the same time.

## Discussion

HT is a complication of acute ischemic stroke (16). However, the risk factors and predictive factors for HT of ischemic stroke are unclear. Previous studies identified many risk factors for HT after intravenous thrombolysis, including smoking, diabetes mellitus, atrial fibrillation, hypertension, serum glucose levels, and past history of stroke (17). Moreover, some molecules in serum can predict HT in patients treated with intravenous thrombolysis (15).

In this study, we found that age ≥68 years, smoking, atrial fibrillation, NIHSS score before thrombolysis ≥17, and high systolic pressure 2 h after thrombolysis were risk factors for HT after rt-PA intravenous thrombolysis. Our results showed that Systolic pressure before thrombolysis did not result a significant predictor, whereas higher systolic pressure 2 h after thrombolysis resulted associated with an increased risk of HT. These findings may suggest the influence of blood pressure fluctuations on stroke outcome and disease course and control of both absolute blood pressure levels and blood pressure variability may be therapeutic targets (18–20).

Our study demonstrated that low serum levels of caveolin-1 and caveolin-2 were associated with HT after rt-PA treatment. Caveolin-1, caveolin-2, and caveolin-3 comprise the caveolin family. We did not detect the caveolin-3 level in serum because it is specifically expressed in skeletal muscles (21, 22). It was reported that low serum levels of caveolin-1 is an important predictor of HT after r-tPA administration (15). But because of the small sample size, further research is needed to validate their result. Our study confirmed previous work and got some new finding. Our results showed that the non-hemorrhage group of patients presented significantly higher caveolin-1 and caveolin-2 levels than the hemorrhage group of patients after intravenous thrombolysis treatment. Caveolin-1 and caveolin-2 can modulate the permeability of endothelium and blood-brain barrier after stroke and inhibit the release of free radicals. Accordingly, it may be assumed that they interact synergistically with the inflammatory cascade following acute cerebral insult. In this regard, it is worth to notice that serum inflammatory biomarkers have been shown to play role in hematoma growth and edema formation (23–25). Furthermore, there is a physiologic relevant role of caveolin-1 in the effect of endothelial nitric oxide synthase activation and cardiovascular disease (26). It would be interesting to explore the association between inflammatory peripheral biomarkers obtained after thrombolysis and both the levels of caveolins and the occurrence of HT. This would allow to provide more insights on the pathophysiology of HT and offer the opportunity to identify easy obtainable markers to be used in clinical practice.

A previous study reported that caveolin-1 can inhibit the release of free radicals and the activation of MMPs, thereby preventing endothelial disruption. High MMP activity and hyperpermeable microvascular system are observed in caveolin-1-deficient mice. Moreover, administering the caveolin-1 scaffolding domain or nitric oxide synthase inhibitors can alleviate the symptoms of caveolin-1-deficient mice (27–30). Although caveolin-2 is heterooligomerized and coexpressed with caveolin-1 in endothelial cells, adipocytes, and many other cell types, research on caveolin-2 is not extensive. Our study demonstrated that both caveolin-1 and caveolin-2 are associated with HT after rt-PA administration. One limitation of this study is its single-center, retrospective design, which may have resulted in selection bias. Moreover, high admission glucose levels, onset-to-treatment time, and arterial stiffness are also possible further factors that can contribute to the occurrence of hemorrhagic transformation (31, 32). These factors are not covered in this study.

In summary, our study showed that caveolin-1 and caveolin-2 can be predictors of HT after rt-PA administration. The analysis of several biomarkers at the same time can improve diagnostic accuracy. Our results give support to further research which aimed at validating this biomarker as well as determining whether its joint analysis with other previously reported HT biomarkers improve their predictive capacity.

## Data Availability Statement

All datasets generated for this study are included in the article/supplementary material.

## Ethics Statement

The studies involving human participants were reviewed and approved by the Human Ethics Committees of Chengde Medical College. The patients/participants provided their written informed consent to participate in this study.

## Author Contributions

Z-jD designed the research. FS, HL, H-xF, C-bL, and X-jG performed the research. FS, HL, X-xZ, JZ, ZM, and Y-jG analyzed data. All authors have read and approved the final manuscript.

## Conflict of Interest

The authors declare that the research was conducted in the absence of any commercial or financial relationships that could be construed as a potential conflict of interest.

## References

[1] Guéniat J Brenière C Graber M Garnier L Mohr S Giroud M. Increasing burden of stroke: the dijon stroke registry (1987-2012). Neuroepidemiology. (2018) 50:47–56. 10.1159/000486397 29393231

[2] Jickling GC Manolescu BN. Breaking down barriers to identify hemorrhagic transformation in ischemic stroke. Neurology. (2012) 79:1632–3. 10.1212/WNL.0b013e31826e9b9d 22993281

[3] Knight RA Barker PB Fagan SC Li Y Jacobs MA Welch KM. Prediction of impending hemorrhagic transformation in ischemic stroke using magnetic resonance imaging in rats. Stroke. (1998) 29:144–51. 10.1161/01.STR.29.1.144 9445344

[4] Paciaroni M Agnelli G F Ageno W Alberti A Lanari A. Early hemorrhagic transformation of brain infarction: rate, predictive factors, and influence on clinical outcome: results of a prospective multicenter study. Stroke. (2008) 39:2249–56. 10.1161/STROKEAHA.107.510321 18535273

[5] Beslow LA Smith SE Arastoo V Licht DJ Kasner SE Favilla CG. Hemorrhagic transformation of childhood arterial ischemic stroke. Stroke. (2011) 42:941–6. 10.1161/STROKEAHA.110.604199 21350202 PMC3066279

[6] Strbian D Sairanen T Meretoja A Pitkäniemi J Putaala J Salonen O. Patient outcomes from symptomatic intracerebral hemorrhage after stroke thrombolysis. Neurology. (2011) 77:341–8. 10.1212/WNL.0b013e3182267b8c 21715707

[7] Castellanos M Serena J. Applicability of biomarkers in ischemic stroke. Cerebrovasc Dis. (2007) 24:7–15. 10.1159/000107374 17971634

[8] Castellanos M Sobrino T Millan M Garcia M Arenillas J Nombela F. Serum cellular fibronectin and matrix metalloproteinase-9 as screening biomarkers for the prediction of parenchymal hematoma after thrombolytic therapy in acute ischemic stroke: a multicenter confirmatory study. Stroke. (2007) 38:1855–9. 10.1161/STROKEAHA.106.481556 17478737

[9] Rothberg KG Heuser JE Donzell WC Ying Y-S Glenney JR Anderson RGW. Caveolin, a protein component of caveolae membrane coats. Cell. (1992) 68:673–82. 10.1016/0092-8674(92)90143-Z 1739974

[10] Scherer PE. Expression of caveolin-3 in skeletal, cardiac, and smooth muscle cells. Caveolin-3 is a component of the sarcolemma and co-fractionates with dystrophin and dystrophin-associated glycoproteins. J Biol Chem. (1996) 271:15160–5. 10.1074/jbc.271.25.15160 8663016

[11] Zhang S Liu Y Zhao Z Xue Y. Effects of green tea polyphenols on caveolin-1 of microvessel fragments in rats with cerebral ischemia. Neurol Res. (2010) 32:963–70. 10.1179/016164110X12700393823570 20444327

[12] Liu LB Xue YX Liu YH. Bradykinin increases the permeability of the blood-tumor barrier by the caveolae-mediated transcellular pathway. J Neurooncol. (2010) 99:187–94. 10.1007/s11060-010-0124-x 20146088

[13] Zhao LN Yang ZH Liu YH Ying HQ Zhang H Xue YX. Vascular endothelial growth factor increases permeability of the blood–tumor barrier via caveolae-mediated transcellular pathway. J Mol Neurosci. (2011) 44:122–9. 10.1007/s12031-010-9487-x 21193965

[14] Xu L Guo R Xie Y Ma M Ye R Liu X. Caveolae: molecular insights and therapeutic targets for stroke. Expert Opin Ther Targets. (2015) 19:633–50. 10.1517/14728222.2015.1009446 25639269

[15] Castellanos M van Eendenburg C Gubern C Kádár E Huguet G Puig J. Low levels of caveolin-1 predict symptomatic bleeding after thrombolytic therapy in patients with acute ischemic stroke. Stroke. (2018) 49:1525–7. 10.1161/STROKEAHA.118.020683 29712879

[16] Yaghi S Willey JZ Cucchiara B Goldstein JN Gonzales NR Khatri P. Treatment and outcome of hemorrhagic transformation after intravenous alteplase in acute ischemic stroke: a scientific statement for healthcare professionals from the American heart association/American stroke association. Stroke. (2017) 48:e343–61. 10.1161/STR.0000000000000152 29097489

[17] Wang BG Yang N Lin M Lu B Analysis of risk factors of hemorrhagic transformation after acute ischemic stroke: cerebral microbleeds do not correlate with hemorrhagic transformation. Cell Biochemi Biophys. (2014) 70:135–42. 10.1007/s12013-014-9869-8 24691925

[18] Divani AA Liu X Napoli MD Lattanzi S Petersen A Blood pressure variability predicts poor in-hospital outcome in spontaneous intracerebral hemorrhage. Stroke. (2019) 50:2023–9. 10.1161/STROKEAHA.119.025514 31216966

[19] Arima H Heeley E Delcourt C Hirakawa Y Wang X Woodward M. Optimal achieved blood pressure in acute intracerebral hemorrhage: INTERACT2. Neurology. (2015) 84:464–71. 10.1212/WNL.0000000000001205 25552575 PMC4336065

[20] Buratti L Cagnetti C Balucani C Viticchi G Falsetti L Luzzi S. Blood pressure variability and stroke outcome in patients with internal carotid artery occlusion. J Neurol Sci. (2014) 339:164–8. 10.1016/j.jns.2014.02.007 24582284

[21] Xie H Lu WC Inhibition of transient receptor potential vanilloid 4 decreases the expressions of caveolin-1 and caveolin-2 after focal cerebral ischemia and reperfusion in rats. Neuropathology. (2018) 38:337–46. 10.1111/neup.12469 29665111

[22] Babak R Bo WX Engelman JA Michela B Guy L Lan ZX. Caveolin-2-deficient mice show evidence of severe pulmonary dysfunction without disruption of caveolae. Mol Cell Biol. (2002) 22:2329–44. 10.1128/MCB.22.7.2329-2344.2002 11884617 PMC133690

[23] Di Napoli M Slevin M Popa-Wagner A Singh P Lattanzi S Divani AA. Monomeric C-reactive protein and cerebral hemorrhage: from bench to bedside. Front Immunol. (2018) 9:1921. 10.3389/fimmu.2018.01921 30254628 PMC6141664

[24] Lattanzi S Napoli MD Ricci S Divani AA. Matrix metalloproteinases in acute intracerebral hemorrhage. Neurotherapeutics. (2020) 17:484–96. 10.1007/s13311-020-00839-0 31975152 PMC7283398

[25] Lattanzi S Brigo F Trinka E Cagnetti C Napoli MD Silvestrini M. Neutrophil-to-lymphocyte ratio in acute cerebral hemorrhage: a system review. Transl Stroke Res. (2019) 10:137–45. 10.1007/s12975-018-0649-4 30090954

[26] Guanghong J James SR. Caveolin-1 in cardiovascular disease: a double-edged sword. Diabetes. (2015) 64:3645–7. 10.2337/dbi15-0005 26494216 PMC4613981

[27] Schubert W Frank PG Woodman SE Hyogo H Cohen DE Chow CW. Microvascular hyperpermeability in caveolin-1 (–/–) knock-out mice: treatment with a specific nitric-oxide synthase inhibitor,l-name, restores normal microvascular permeability in cav-1 null mice. J Biol Chem. (2002) 277:40091–8. 10.1074/jbc.M205948200 12167625

[28] Gu Y Zheng G Xu M Li Y Chen X Zhu W. Caveolin-1 regulates nitric oxide-mediated matrix metalloproteinases activity and blood-brain barrier permeability in focal cerebral ischemia and reperfusion injury. J Neurochem. (2015) 120:147–56. 10.1111/j.1471-4159.2011.07542.x 22007835

[29] Gu Y Dee C Shen J. Interaction of free radicals, matrix metalloproteinases and caveolin-1 impacts blood-brain barrier permeability. Front Biosci. (2011) 3:1216. 10.2741/222 21622267

[30] Zhang J Wusheng Z Lulu X Qinqin C Hao Z Huaiming W. Lower serum caveolin-1 is associated with cerebral microbleeds in patients with acute ischemic stroke. Oxidat Med Cell Long. (2016) 2016:9026787. 10.1155/2016/9026787 27119011 PMC4826928

[31] Paciaroni M Agnelli G Caso V Corea F Ageno W Alberti A. Acute hyperglycemia and early hemorrhagic transformation in ischemic stroke. Cerebrovasc Dis. (2009) 28:119–23. 10.1159/000223436 19506370

[32] Acampa M Camarri S Lazzerini PE Guideri F Martini G. Increased arterial stiffness is an independent risk factor for hemorrhagic transformation in ischemic stroke undergoing thrombolysis. Int J Cardiol. (2017) 243:466–70. 10.1016/j.ijcard.2017.03.129 28747037

